# Fellowships, Grants, & Awards

**Published:** 2007-02

## The Genes, Environment, and Development Initiative (U01)

This Request for Applications (RFA) from the National Institute on Drug Abuse (NIDA) and the National Cancer Institute (NCI) launches the Genes, Environment, and Development Initiative (GEDI). The GEDI will support research using existing research samples to investigate main effects, correlations, and interactions among genetic, environmental, and developmental factors in the etiology of substance abuse and related phenotypes in humans. The term “substance abuse” as used in this RFA refers broadly to several different but related concepts including substance use (quantity, frequency, patterns, trajectories) and substance abuse and dependence as defined by diagnostic criteria. While it is recognized that these concepts may not be interchangeable, the term “substance abuse” is used for the purposes of fluency. The term “substance” refers primarily to nicotine, alcohol, cannabis, cocaine, stimulants, and opiates, but also includes the entire range of licit and illicit substances of potential addiction. The term “related phenotypes” refers to constructs that have been shown empirically to convey risk for substance abuse, such as behavioral disinhibition. “Developmental research” refers to the study of progressive change that occurs as humans move through the lifecourse.

The National Institute on Mental Health (NIMH) has an interest in applications where the research contributes to knowledge about the interplay of genetic, environmental, and developmental factors in the etiology of adverse outcomes; however, no funds will be committed at this time.

Prior lines of research have established that genetic, environmental, and developmental factors all contribute to vulnerability to substance abuse and related phenotypes. Both animal and human studies have demonstrated genetic influences on substance abuse, with heritability estimates ranging from 40 to 60%, and promising candidate loci and genes for substance abuse have been identified. Genetic influences have also been shown for many related phenotypes, and some (e.g., externalizing disorders) share genetic factors with substance abuse. As with other complex disorders, it is likely that multiple genes of small effects contribute to vulnerability to substance abuse and related phenotypes, and that environmental conditions moderate genetic influence. A wide variety of environmental variables have been correlated with substance abuse and related phenotypes, including *in utero* exposures, parenting, trauma and stress, peer influences and/or neighborhood and societal factors. Many of these correlations have been very strong and frequently replicated, forming the basis for numerous preventive intervention studies with youth at high risk due to individual and familial factors in combination with the social context over the course of development. The role of developmental factors is highlighted not only by the fact that the human brain is an organ that continues to develop until the mid-twenties, but also by the changes in substance abuse patterns associated with significant developmental shifts, including childhood transitions, pubertal transitions (often coinciding with substance use initiation), transitions to greater independence after high school (associated with increased substance use and shifts from use to dependence), and adult role transitions such as marriage, parenting, and full time employment (associated with decreased substance abuse). Although less well understood, developmental timing of substance abuse has been assumed to be important as well, with different outcomes (e.g., addiction) expected depending on when certain events (e.g., onset of substance use) occur over the life course. Expression patterns of genes associated with substance abuse change over the course of development, either through epigenetic modifications that are developmental and tissue specific, or through genetic variation that may affect gene expression during certain stages of neurodevelopment. For instance, animal studies have shown that exposure to nicotine during adolescence, compared to adulthood, predicts greater drug-seeking behavior and neurologic change in critical areas of the brain. Similarly, human research has shown that prenatal exposure to nicotine increases the risk of nicotine dependence during adulthood.

While these lines of research have made important contributions to our understanding of substance abuse, they each have limitations in delineating the etiology of substance abuse and related phenotypes, and they can benefit from integration and interaction with the others. The inferential power of environmental and developmental studies can be significantly enhanced by the inclusion of analyses of gene–environment correlations, that is, genetic influences on environmental exposures. For example, by accounting for family history or genetic susceptibility to substance abuse, studies of prenatal-exposure effects on later behavior can better determine the role of passive gene–environment correlation, which in turn can help distinguish between exposure effects and family history as the source of behavioral outcomes. Similarly, studies of peer influences or parenting behaviors in adolescence will be strengthened by taking into account passive, active, and evocative gene–environment correlations, which can help to clarify the role of the adolescent’s choices and behaviors in eliciting these environmental factors. In addition, molecular genetics studies seeking to identify specific susceptibility genes can be greatly enhanced in their power and accuracy by attending to interactions with environmental and developmental factors that may affect the expression of these genes.

Over many years, NIDA, other NIH institutes, and other organizations have funded numerous high-quality longitudinal and developmental studies that contain a wealth of data from individuals who are at risk for, or are in the course of development, progression, and desistance of, substance abuse and related phenotypes. A valuable variety of variables associated with these outcomes has been measured on diverse samples across multiple domains and time points, characterizing psychopathology, temperament, familial relations and practices, peer relations and characteristics, school factors, and broader environmental influences, as well as biological variables including *in utero* and toxic exposures, endophenotypes, and neurocognitive structure and function. In addition, the many randomized intervention trials that have been conducted over the years provide opportunities for strong inferences about the role of social environment in causality by focusing on presumed etiologic social factors and examining covariance of the social environment and adjustment over time. The GEDI seeks to build on this substantial public investment by soliciting applications that integrate environmental and developmental variables with genotypic information to permit comprehensive model building and hypothesis testing for determining genetic, environmental, and developmental contributions to substance abuse and related phenotypes.

Applications qualifying for submission under this FOA must rely exclusively on existing studies of human subjects; new data collection is permitted only for the purpose of obtaining blood from subjects so that DNA and cryopreserved lymphocytes can be generated as a renewable resource and stored. Studies with DNA already collected are required to demonstrate that their DNA is a publicly available sharable resource, with a clear process for access to the samples by other qualified investigators. If the DNA samples are stored at a site other than the NIDA repository, plans for electronically depositing clinical data, phenotypic data, and genetic data into the NIDA Repository for access and sharing need to be provided. If the collection of new data is required to evaluate the disorder (e.g., for studies where individuals are just entering the period of risk for substance abuse), plans for collecting these data through another funding mechanism should be included. Data sets drawn from studies that involve an intervention trial are appropriate for this RFA; these proposals should address the approach to handling intervention effects when exploring etiologic questions. If intervention data sets are proposed for use to infer causal influence (i.e., to explore whether intervening on mediating variables changed outcomes in expected ways, thus demonstrating the strength of the mediator in “causing” the outcome of interest), then investigators must demonstrate successful randomization procedures and sufficient power to detect and explicate observed short- and long-term environmental intervention effects in the sample. Proposals requesting support to collect new data other than DNA under this RFA research mechanism or to conduct research using animal models will be considered nonresponsive and returned to the applicant.

Established studies/samples eligible for analyses within GEDI applications must include the following characteristics: 1) subjects who are in or through the period of risk for substance abuse and related phenotypes; 2) subjects who have provided the appropriate consent for or can be recontacted for additional consent for GEDI research (e.g., to permit data sharing, blood collection, genetic analyses, etc.); 3) DSMIIIR or DSMIV diagnoses as well as other categorical and/or quantitative measures of substance abuse and related phenotypes with demonstrated heritability; 4) environmental data relevant to substance abuse and related phenotypes; 5) data waves spanning at least two developmental periods, such as *in utero*, infancy, early childhood, late childhood, early adolescence, late adolescence, emerging adulthood, and/or adulthood.

Applications should present a rationale that carefully balances important substantive, methodologic, and budgetary issues. In particular, applications should fully address each of the following points, where applicable: 1) Sample selection (e.g., particularly informative subsamples and subgroups, understudied valuable populations); 2) Research design (e.g., genetically informative, birth cohort, multigenerational, selection by prenatal exposure, randomized intervention); 3) Statistical power to detect the proposed main effects, correlations, and interactions. In cases where individual data sets lack sufficient power to address study aims, data from multiple sites may be pooled; 4) Where pooling is planned, the application must include procedures for combining data. In all cases where multiple data sets are used, pilot data must be provided within the application to demonstrate reasonable compatibility of data across studies and evidence of feasibility of multisample analyses; 5) In cases where multiple data sets are used, evidence of collaboration among sites must be shown; 6) Generalizability of results [i.e., population(s) to which findings can be applied]; 7) Phenotype(s) selection (e.g., specific heritable phenotypes, related phenotypes, endophenotypes); 8) Number and timing of assessments (e.g., multiple waves ranging from pre- to post-substance exposure, from substance use initiation to disorder onset, or from disorder onset to remission); 9) Selection and quality of assessments (e.g., diagnostic and/or symptom-based scales, measurement of salient individual and environmental factors); 10) comorbidity (e.g., psychiatric, polysubstance, physical illness); 11) Timeline to collect and submit clean data (i.e., genetic, environmental, developmental data) to the NIDA Repository; 12) Consent by subjects to allow qualified investigators to use and analyze data and DNA; 13) In view of the technical complexity of the science called for in this announcement, the GEDI requires transdisciplinary alliances among researchers with appropriate breadth and depth of expertise. Applications should have a substantial commitment of personnel for statistical genetics and bioinformatics, with detailed plans for data collection, storage, communication, integration, analyses, and dissemination; 14) Proposals are required to include detailed plans and timelines for replicating initial GEDI findings within the 5-year award period. Plan(s) for replicating should be fully explained and justified given that there are many approaches for duplicating research findings (e.g., reproducing results between randomly selected subsamples samples from a single data set, replicating findings across multiple data sets with comparable measures, pooling data from multiple data sets and replicating findings from randomly selected subsamples); 15) Genotyping and analytic methodologies: Applicants should note that the genotyping platform ultimately chosen will be dependent upon an agreement within the GEDI Steering Committee (described in Section 6.2.A.3) to ensure consistent and standard genotypes and allele names. Although the genotyping approaches (candidate genes or whole genome scans, for example) outlined for each funded grant may differ, the GEDI Steering Committee will be expected to provide input on this effort among the cooperative agreements so that a consensus strategy is achieved. Nevertheless, a description and rationale of the platform(s) proposed for genotyping should be included. Applications should address the following: *a*) an explicit rationale for the genotyping approach chosen (candidate gene, linkage analysis, whole genome association); *b*) a plan to implement the genotyping and milestones for achieving the genotyping in the time frame of the funded period. This proposal should thoroughly discuss and justify the plans for genotyping, assessing genotype quality, and depositing the genotypes into the NIDA Repository; *c*) all costs for genotyping, taking into account delivery of the DNA, genotyping SNPs, repeating failed samples, assessing genotype quality, deposition of the data, costs of the genotyping facility preferred. Depending on the GEDI Steering Committee recommendations, the genotyping facility may not be the one initially proposed. Budgets for future years may be contingent on the genotyping strategy chosen. Applicants should be aware that future years of allocation of funds for this component may be changed to reflect the scientific approach chosen and the funds needed; *d*) a detailed analytic plan addressing issues related to the large amount of GEDI data to be generated, such as multiple-testing issues, assessing correlations and complex interactions (gene × environment, gene × development, environment × development, gene × environment × development), impact of population ancestry (stratification), false positives, etc.; *e*) a rationale for single SNP, multiple SNP (e.g., from multiple pathways) and/or haplotype-based analyses; 16) All phenotypic, environmental, developmental, and genotypic data analyzed through GEDI will be made available in de-identified form to the scientific community through the NIDA Repository. All investigators, whether or not they join the NGC, will be required to provide a timeline for submitting cleaned data to the NIDA Repository, and to participate in the NGC biannual meetings in Rockville, MD; travel and accommodations should be included in the budget; 17) All applicants are encouraged to join the NIDA Genetics Consortium (NGC) and to submit blood samples as well as the required genetic, environmental, and developmental data to the NIDA Repository. Funds for up to 15,000 total samples (or 3,000–5,000 per award) for blood processing, DNA extraction, storage, and distribution will be provided for all awards joining the NGC. A budget needs to be presented if > 5,000 samples will be collected. Additionally, a fee structure for distribution of DNA from the NIDA Repository can be found at http://www.nida.nih.gov/about/organization/Genetics/FAQ_NCGS.html and applicants should determine whether additional budgetary allocations are needed for DNA requests exceeding the NIDA Repository allowances. Studies funded under this FOA will not require NIDA Access Committee review; 18) In cases where NGC membership is prohibited (e.g., by laws governing foreign-derived samples) or not chosen, applicants must *a*) demonstrate that genetic, environmental, and developmental data can be submitted to the NIDA repository; *b*) demonstrate that DNA samples have been or will be submitted to a publicly available repository that permits data sharing to qualified researchers; *c*) present the repository’s quality control procedures for processing DNA, cryopreserving or immortalizing blood, and archiving DNA samples; and *d*) describe the repository’s track record of providing data access by qualified investigators.

Close interaction among GEDI Investigators will be required to develop appropriate strategies and tools to design and conduct GEDI research. The awardees will convene as a GEDI Steering Committee, which will include representatives from each of the awards and the NIH, will meet twice a year and participate on monthly conference calls to share information on data resources, methodologies, analytical tools, as well as data and preliminary results. Subcommittees and working groups may be established, such as a genotyping or analysis group. A 4–5 member GEDI Advisory Board will also be created by the GEDI Steering Committee. Costs associated with attending meetings and monthly conference calls should be included in the proposed research budget. An additional budgetary item should include costs to support two of the GEDI Advisory Board members who will convene annually.

The GEDI has the primary goal of elucidating the contribution of genetic, environmental, and developmental factors to the etiology of substance abuse and related phenotypes. Funded research under the GEDI is expected to lead to improved and tailored preventive and treatment interventions for these common and costly outcomes.

GEDI applications must address the interplay of genetic, environmental, and developmental factors related to substance abuse and related phenotypes. Although individual components (e.g., genetic, environmental or developmental or their combination) may be examined within the application, the ultimate goal of the study must integrate these components.

Where possible, investigators are encouraged to explore how relationships differ by sex/gender and race/ethnic group.

Examples of appropriate research topics include, but are not limited to, the following: 1) studies of the role of gene–environment–development interactions in the initiation of drug use and progression to addiction, including but not restricted to studies of the impact of environmental factors in individuals with known genotypes for particular alleles; 2) studies of the role of gene-environment correlation that clarify how individuals select and influence their environments over developmental stages, and to help identify true gene–environment–development interactions; 3) studies employing refined and systematic environmental and developmental measures that identify salient factors (e.g., trauma, peers, siblings) associated with risk for substance abuse, stratified by genotypes associated with substance abuse or related disorders; 4) studies to address the salience of initial drug exposure (e.g., developmental timing, type, amount, context, subjective and objective responses) for subsequent substance abuse risk and course, incorporating the role of genetic risk (genotype; gene–environment correlations and interactions); 5) studies that explore the heterogeneity of genetic, environmental, and developmental contributions to substance abuse for different subgroups, including race/ethnic groups, sex/gender groups, cultural groups, etc.; 6) identification of biomarkers, endophenotypes, and subclinical phenotypes that vary or change over development as a function of environmental and genetic risk; 7) studies distinguishing the genetic, environmental, and developmental factors and their interactions in the vulnerability to substance abuse on specific drugs versus factors contributing to general liability; 8) studies that examine the differential effects of salient developmental (e.g., pubertal timing) and environmental (e.g., stress, peer influences) events interacting with other variables that confer or protect from vulnerability to substance abuse and related phenotypes; 9) studies that explicate the mechanisms by which genes, environment, and development confer risk for substance abuse and related phenotypes (e.g., delinquency, gambling, HIV risk behaviors); 10) studies of the interplay of genetic, environmental and developmental factors on differential patterns of risk associated with comorbid psychiatric disorders, including psychotic and internalizing disorders; 11) studies of the interplay of genetic, environmental, and developmental factors on differential patterns of risk associated with the timing and dosages of prescribed medications, including but not limited to psychostimulants; 12) studies of underlying vulnerability factors and endophenotypes that may mediate genetic risk for substance abuse and related phenotypes, including studies of temperament and neurophysiology; 13) studies that explore the impact of population differences in allele frequencies on gene × environment × development interactions for substance abuse and related phenotypes; 14) research using molecular approaches to study the impact of cumulative, acute, and/or chronic environmental exposures or psychosocial stressors in specific developmental periods; 15) research evaluating environment and development data that include the analysis of combined effects of gene variants from a gene family or cellular pathways involved in addiction; 16) studies of *in utero* drug exposure that identify genetic and environmental factors that discriminate individuals who do and do not develop substance abuse and related phenotypes; 17) studies that explore whether effective interventions focusing on social or other environmental elements work by altering gene expression, or whether genetic variability accounts for why interventions work for some participants and not for others; 18) studies that address the interplay of genetic, environmental, and developmental factors within intervention outcome data sets, taking advantage of the unique opportunity within intervention research to infer causal influence by demonstrating changes in mediators that are related to outcomes of interest; 19) studies of the interplay of genetic, environmental, and developmental factors associated with increased risk for behavioral disorders in offspring exposed to smoking *in utero*; 20) studies of the interplay of genetic, environmental, and developmental factors conferring resilience in individuals at high risk for substance abuse and related outcomes; 21) evaluation of functional polymorphisms that are moderated by environmental and developmental factors; 22) studies analyzing the genetic mechanisms by which environmental factors such as therapeutic compounds, stress, alcohol, and drugs alter the expression of genes, e.g., through changing methylation status; 23) approaches that specifically model combinations of *a*) single SNPs, multiple SNPs, and/or haplotypes and environmental exposures; *b*) single SNPs, multiple SNPs, and/or haplotypes and developmental trajectories; *c*) single SNPs, multiple SNPs, and/or haplotypes and environment and development variables.

NIDA staff will conduct an application information teleconference at a date and time to be determined. This meeting will allow potential applicants to discuss and clarify any issues related to this FOA with NIDA staff. Detailed information about the meeting will be updated and available on the NIDA website (http://www.nida.nih.gov/about/organization/Genetics/index.html), and a set of frequently asked questions and staff responses will be posted. Please submit your questions via email to Dr. Weinberg ( nw46w@nih.gov) at least one day in advance of the teleconference.

This funding opportunity will use the U01 Research Project Cooperative Agreement award mechanism. As an applicant, you will be solely responsible for planning, directing, and executing the proposed project.

This funding opportunity uses the just-in-time budget concepts. It also uses the nonmodular budget format described in the PHS 398 application instructions (see http://grants.nih.gov/grants/funding/phs398/phs398.html). A detailed categorical budget for the “Initial Budget Period” and the “Entire Proposed Period of Support” is to be submitted with the application.

The PHS 398 application instructions are available at http://grants.nih.gov/grants/funding/phs398/phs398.html in an interactive format. Applicants must use the currently approved version of the PHS 398.

For further assistance contact GrantsInfo, 301-435-0714, (telecommunications for the hearing impaired: TTY 301-451-0088), or by e-mail: GrantsInfo@nih.gov.

Applications must be prepared using the most current PHS 398 research grant application instructions and forms. Applications must have a D&B Data Universal Numbering System (DUNS) number as the universal identifier when applying for Federal grants or cooperative agreements. The D&B number can be obtained by calling 866-705-5711 or through the website at http://www.dnb.com/us/. The D&B number should be entered on line 11 of the face page of the PHS 398 form.

The letter of intent receipt date for this RFA is 15 February 2007, with the application receipt date 15 March 2007. The complete version of the RFA is available at http://grants/nih.gov/grants/guide/rfa-files/RFA-DA-07-012.

Contacts: Naimah Weinberg, Division of Epidemiology, Services, and Prevention Research, National Institute on Drug Abuse, 6001 Executive Boulevard, Room 5185, Bethesda, MD 20892-9589 USA, 301-402-1908, fax: 301-443-2636, e-mail: nw46w@nih.gov; Glen D. Morgan, Tobacco Control Research Branch, Behavioral Research Program, Division of Cancer Control and Population Sciences, National Cancer Institute, 6130 Executive Boulevard, Room 4034, Bethesda, MD 20892-7337 USA, 301-496-8585, fax: 301-496-86756, e-mail: gmorgan@mail.nih.gov.

Reference: RFA-DA-07-012.

## International Research Scientist Development Award (IRSDA) [K01]

The International Research Scientist Development Award (IRSDA) provides research opportunities, as well as cutting-edge technical training, in leading developing country institutions for U.S. postdoctoral biomedical, epidemiological, clinical, social, and behavioral scientists who are committed to careers in international health research. The award supports the recipient for a 3- to 4-year period of collaboration with a U.S. mentor and an established developing country mentor. This collaboration should be based on a research project of mutual interest in the context of an ongoing research relationship between the U.S. and foreign mentors. It is expected that this experience will prepare scientists to pursue an independently funded global health research career involving ongoing collaboration with developing country scientists.

The IRSDA is part of the FIC strategy to support research collaborations in developing countries in order to build research capacity to address global health research priorities. The role of the IRSDA is to: 1) attract new research talent to global health research and enhance research collaboration between the U.S. and foreign sites; 2) advance the career paths of exceptional junior U.S. scientists with mentored training in health issues of developing country populations; 3) extend the impact and scope of existing career research and training support for U.S. scientists committed to international research; and 4) stimulate a more effective translation of the results of research on global health problems into public health practice.

With IRSDA support, the investigator will have the opportunity to work closely with an established foreign scientist in the developing world and a U.S. investigator, who are involved in collaborative research. The applicant will conduct research and receive training at both the United States and developing country institutions. It is expected that these awards will serve to forge collaborative relationships between established, developing country researchers and outstanding U.S. junior scientists who are potential leaders of basic, clinical and behavioral/social health research programs in the U.S. Collaborations are expected to lead to advances that will reduce the impact of global health problems and narrow the gap in health disparities between developed and developing countries.

All career development proposals must be tailored to meet the individual needs of the candidate. The specific career development and research training activities proposed in the application may be new to the candidate or an extension of the candidate’s prior research, but should focus on global health concerns which include, but are not limited to, infectious diseases, nutrition, chronic/degenerative conditions, trauma/injury and mental health disorders. Basic laboratory, behavioral/social and clinical biomedical research will be supported in clinical, field or laboratory settings.

The candidate must devote a minimum of 75% of full professional effort to the goals of this award, including significant time devoted to research in the chosen developing country. Applicants for initial awards must agree to spend a minimum of 50% of the project period of the grant at the foreign research site for at least 3 months per year. Applicants for a competitive 3-year renewal mentored career development award must agree to spend a minimum of 1 year of the total project period at the foreign research site for at least 3 months per year.

The candidate and the U.S. and foreign mentors are jointly responsible for the preparation of the plan for career development. The applicant must justify the need for this award and provide a convincing case that the proposed period of support will substantially enhance his or her career as an independent investigator in global health research. The sponsoring institution must be able to demonstrate a commitment to the development of the candidate as a productive, independent investigator. FIC recognizes that there will be significant differences in the applicants, U.S. and foreign institutional environments, U.S. and foreign mentors’ backgrounds, and approaches to international research collaboration among applications. Therefore, applicants should clearly define specific research and training goals related to each mentor and each institution, methods to achieve the goal of an independent research career in global health, and specific measurable objectives to enable assessment of the proposed project.

This funding opportunity will use the NIH career development (K01) award mechanism (PA-06-001). Please note that this K01 award has some important differences from the K01 award described in PA-06-001. These include: 1) the original award period is for 3 to 4 years; 2) a competitive renewal mentored career development award is possible for up to 3 years; 3) Both a U.S. and a foreign mentor are required and must share a research collaboration; and 4) requirement of significant time spent in the foreign country.

This funding opportunity uses the just-in-time budget concepts. It also uses the nonmodular budget format described in the PHS 398 application instructions (see http://grants.nih.gov/grants/funding/phs398/phs398.html). A detailed categorical budget for the “Initial Budget Period” and the “Entire Proposed Period of Support” is to be submitted with the application.

Initial awards are for 3 to 4 years. Competitive renewal awards are allowable for awardees who have obtained a tenure-track position and additional mentored support can be for up to 3 years. Awards are not transferable from one principal investigator to another.

The PHS 398 application instructions are available at http://grants.nih.gov/grants/funding/phs398/phs398.html in an interactive format. Applicants must use the currently approved version of the PHS 398. For further assistance contact GrantsInfo, 301-435-0714, (telecommunications for the hearing impaired: TTY 301-451-0088, or by e-mail: GrantsInfo@nih.gov.

Applications must be prepared using the most current PHS 398 research grant application instructions and forms. Applications must have a D&B Data Universal Numbering System (DUNS) number as the universal identifier when applying for Federal grants or cooperative agreements. The D&B number can be obtained by calling 866-705-5711 or through the web site at http://www.dnb.com/us/. The D&B number should be entered on line 11 of the face page of the PHS 398 form.

The letter of intent dates for this PAR are 14 December 2007, and 16 December 2008, with the application receipt dates 16 January 2007, 2008, and 2009. The complete version of this PAR is available at http://grants.nih.gov/grants/guide/pa-files/PAR-07-014.html.

Contact: Barbara Sina, Division of International Training and Research, Fogarty International Center, National Institutes of Health, 31 Center Drive, Room B2C39, Bethesda, MD 20892-2220, 301-496-1653, fax: 301-402-0779, e-mail: sinab@mail.nih.gov.

Reference: PAR-07-014.

